# Long Drainers After Abdominoplasty: A Risk Analysis

**DOI:** 10.1007/s00266-025-04773-4

**Published:** 2025-03-25

**Authors:** Margarida Alves, Margarida Mendes, Rita Valença-Filipe, Marco Rebelo, Helena Peres, António Costa-Ferreira

**Affiliations:** 1 Centro Hospitalar de Setúbal, Setúbal, Portugal; 2 Department of Surgery and Physiology, Faculty of Medicine, Porto University, Porto, Portugal; 3 Plastic Reconstructive and Aesthetic Surgery Department, São João University Hospital, Porto, Portugal; 4 Plastic Surgery Department, Portuguese Institute of Oncology of Porto, Porto, Portugal; 5 Science Faculty, Porto University, CIIMAR, Porto, Portugal

**Keywords:** Classical abdominoplasty, Long drainers, Risk factors, Suction drains

## Abstract

**Background:**

Suction drains are still one of the most accepted strategies for lowering abdominoplasty postoperative complications. Long periods with drains have been reported after a full abdominoplasty and are associated with patient discomfort, limited mobility, and slower recovery. The clinical profile of Long drainers has yet to be investigated.

**Objective:**

Identify risk factors that increase the number of days with drains.

**Methods:**

A single-center retrospective observational study of patients submitted to classical abdominoplasty was performed. Patients were allocated to one of two groups: Long drainers (≥ 6 days with drains) and Short drainers (< 6 days with drains). Several variables were determined: age, sex, body mass index, medical comorbidities (hypertension and *diabetes mellitus*), previous surgical procedures, specimen weight, time to suction drain removal, and drain output.

**Results:**

In total, 418 patients were included in this study, and 36% were Long drainers. There was a statistically significant difference between groups regarding total drain output, time until drain removal, body mass index, previous bariatric procedures, and specimen weight, with lower values for Short drainers. No significant differences were found in age, sex, arterial hypertension, *diabetes mellitus*, and previous abdominal surgery. Specimen weight ≥ 750 g, body mass index ≥ 28 kg/m^2^, and previous bariatric surgery accounted for 75% of Long drainers and increased Long drainer risk by 3.5 times, 3.0 times, and 2.6 times, respectively.

**Conclusion:**

The high-risk profile for long drainage after classical full abdominoplasty is a body mass index ≥ 28 kg/m^2^, previous bariatric procedure, and specimen weight ≥ 750 g. These characteristics may justify using surgical strategies for Long drainer prevention, such as quilting sutures or Scarpa sparing abdominoplasty.

**Level of Evidence IV:**

This journal requires that authors assign a level of evidence to each article. For a full description of these Evidence-Based Medicine ratings, please refer to the Table of Contents or the online Instructions to Authors  www.springer.com/00266.

## Introduction

Abdominoplasty is frequently performed, has evolved significantly over the years, but continues to imply a considerable risk of complications [1]. A total complication rate of 39% and a seroma rate of 23% were reported based on a meta-analysis of fifteen studies [2]. Seroma consists of a benign fluid collection in the abdominal wall with a multifactorial formation [3, 4].

One of the first strategies used to prevent seroma, and still one of the most accepted, is using postoperative suction drains [4–7]. Most surgeons use two drains, with their orientation and exit points varying according to the surgeon’s preference. It is desirable to use suction drains for the shortest time possible due to the risk of infection, restriction to mobility, and patient discomfort. Criteria for drain removal are usually volume-controlled: Less than 30 ml of aspirate collected in each drain over 24 hours is the most consensual option [5, 8]. Long periods with drains are frequently reported by several authors when using a classical abdominoplasty technique. In two studies, Matarasso [6] and Kim [9] reported an average time until drain removal of 8 days. In the latter study, some patients presented with a maximum period of drain use of 28 days [9]. Neaman also reported long periods with drains in a study involving more than one thousand classical abdominoplasties: The average period of drain use was 18 days [1]. This large study involved five different surgeons, all used suction drains with the following average drain use: surgeon A 11 days, surgeon B 12 days, surgeon C 17 days, surgeon D 10 days, surgeon E 17 days, and surgeon F 14 days. Previously, we reported average periods with drains after a classical full abdominoplasty of 6 days and a maximum time of 21 days [10]. Another study on massive weight loss patients after bariatric surgery showed higher values after a classical abdominoplasty: mean time to suction drain removal of 10 days and maximum time of 45 days [11].

Patients who require extended periods with drains remain a concern, and there is a lack of scientific data to predict who these patients may be. The purpose of this study was to retrospectively analyze patients submitted to a classical abdominoplasty to conclude about risk factors and patient characteristics that contribute to a higher number of days with drains.

## Patients and Methods

A retrospective observational study of all patients who underwent a classical full abdominoplasty between November 2008 and July 2015 in a single institution (Plastic Reconstructive and Aesthetic Surgery Department, São João University Hospital) was performed. The Ethical Committee approved the study.

All patients included in this study underwent a full abdominoplasty with umbilical transposition. Five fully trained plastic surgeons were involved and performed all the surgeries. Preoperative surgical markings are well known and have been previously described elsewhere [12–14]. Preoperative care included subcutaneous enoxaparin (40 mg/day during hospital stay starting at least 2 hours before surgery) and broad-spectrum intravenous antibiotics. Abdominoplasty was performed according to the following principles: low transverse abdominal incision, undermining of the skin and subcutaneous tissue to the costal margins on the plane of the muscular fascia, tightening of the abdominal musculature with correction of rectus muscle diastasis, resection of redundant abdominal skin and subcutaneous tissue, umbilical repositioning, and skin closure. As a classical abdominoplasty, a dissection plane over deep fascia was used without tumescent infiltration, liposuction of the upper abdominal flap, progressive tension sutures, or quilting sutures. Liposuction was limited to the flanks. No additional procedures were done. Compression garments were routinely used and applied in the operating room, and two closed suction drains were placed on each patient. Drains were never removed in the first 24 h, and only when the patient was ambulatory, the drain output in each drain was less than or equal to 30ml collected over 24 h. Patients were discharged from the hospital after drain removal and were instructed to use compression garments and refrain from strenuous activity for at least six weeks after surgery. The average follow-up was two years.

A total of 418 patients were included in the study. The variables taken into account were obtained from a retrospective chart review and were age, sex, body mass index (BMI), medical comorbidities (hypertension and *diabetes mellitus*), previous abdominal surgeries, previous bariatric procedures, weight of the surgical specimen, time to drain removal, and daily and total volume of drain output.

## Statistical Analysis

A two-step cluster analysis, using likelihood distance and Schwarz’s Bayesian criterion as criteria groupings, was done as an exploratory tool to reveal natural groups within the patient’s group. BMI, arterial hypertension, *diabetes mellitus*, previous bariatric surgery, previous abdominal surgery, and specimen weight were used as classification variables. Due to profile similarities, two clusters were created according to the time until drain removal. Cluster 1 and Cluster 2 represent 52.2% and 47.8% of all patients. The most important predictor for cluster membership was time to drain removal: Cluster 1 grouped 100% of the patients with time to drain removal lower than six days, and cluster 2 grouped 75.1% of the patients with time to drain removal higher or equal to 6 days (Table 1). Based on this analysis, the patients were divided into two groups: Long drainers (≥6 days with drains) and Short drainers (< 6 days with drains).

**Table 1:** Two-step cluster analysis of all patients (*N*= 418)

Criteria in order of importance	Cluster 1 52.2%	Cluster 2 47.8%
Importance, 1		
Days until drain removal, frequency	< 6 days (100%)	≥ 6 days (75.1%)
Importance, 0.39		
Total volume drain, average ml	303.6	854.6
Importance, 0.24		
Specimen weight, average g	1032.9	1554.2
Importance, 0.23		
Arterial hypertension, frequency	No (100 %)	No (77.3%)
Importance, 0.23		
Body mass index, average kg/m^2^	25.9	28.9
Importance, 0.22		
Previous bariatric surgery; frequency	No (100 %)	No (78.4 %)
Importance, 0.09		
Diabetes mellitus, frequency	No (100 %)	No (90.8 %)
Importance, 0.06		
Previous abdominal surgery, frequency	Yes (61.9 %)	Yes (77.3 %)

Continuous variables of both groups were analyzed by running t-test and Mann–Whitney U tests, and categorical variables by Chi-squared test. A multivariate logistic model was developed to estimate the relative risk and its 95% confidence intervals of arterial hypertension, *diabetes mellitus*, previous bariatric surgery, body mass index (< 28 or ≥28 kg/m [2]), and specimen weight (< 750 or ≥ 750 g) to predict higher time to drain removal (< 6 or ≥ 6 days). Hosmer–Lemeshow was computed to test for goodness of fit for logistic regression models.

Links between time to drain removal and arterial hypertension, *diabetes mellitus*, previous bariatric surgery, body mass index (< 28 or ≥ 28 kg/m^2^), and specimen weight (< 750 or ≥750 g) were explored by principal component analysis (PCA).

Statistical analysis was performed using SPSS for Windows version 25.0 (SPSS, Inc., Chicago, Ill.).

## Results

After the cluster analysis, the 418 patients submitted to classical abdominoplasty and included in this study were divided into Long drainers (n= 151; 36%) and Short drainers (n= 267; 64%).

The general characteristics of both groups are summarized in Table 2. The average BMI was significantly higher in Long drainers than in Short drainers (28.7 *vs* 26.5 kg/m^2^, *p* < 0.0001). The incidence of previous bariatric surgery was also significantly different (15.9% *vs* 6.7%, *p* < 0.003). The mean specimen weight was significantly higher in Long drainers, with an increase of 40% relative to Short drainers (1568.4 *vs* 1123.9 g, *p* < 0.001). Significant differences were also found in the time to drain removal and total drain output.

**Table 2:** General characteristics of short and Long drainers (*N* = 418)

	Short drainers (< 6 days) (*N* = 267)	Long drainers (≥ 6 days) (*N* = 151)	*P* value*
Age, years			
Mean ± SD Range	39.6 ± 8.7 (21–64)	42 ± 10.5 (14–67)	NS
BMI, kg/m^2^			
Mean ± SD Range	26.5 ± 3.7 (19.1–40.4)	28.7 ± 4.7 (19.1–52.3)	0.0001
Female			
Total number (%)	266 (99.6%)	148 (98.0%)	NS
Arterial hypertension			
Total number (%)	25 (9.4%)	22 (14.6%)	NS
Diabetes mellitus			
Total number (%)	8 (3%)	10 (6.6%)	NS
Previous bariatric surgery			
Total number (%)	18 (6.7%)	24 (15.9%)	0.003
Previous abdominal surgery			
Total number (%)	176 (65.9%)	107 (70.9%)	NS
Specimen weight, g			
Mean ± SD Range	1123.9 ± 573.0 (190–4280)	1568.4 ± 847.4 (250–5700)	0.0001
Time until drain removal, days			
Mean ± SD Range	3.8 ± 0.9 (2–5)	8.8 ± 4.8 (6–45)	0.0001
Total drain output, ml			
Mean ± SD Range	306.8 ± 151.5 (60–900)	1029.4 ± 792.5 (230–4910)	0.0001

BMI, body mass index; NS, nonsignificant (*p* ≥ 0.05)

*The incidence of the female sex, arterial hypertension, diabetes mellitus, previous bariatric surgery, and previous abdominal surgery between both groups were compared using the Chi-squared test. Time to drain removal between both groups was compared using the Mann–Whitney U test. The other variables were compared using the *t*-test

There were no statistically significant differences between groups regarding age, sex, arterial hypertension, *diabetes mellitus*, and previous abdominal surgeries.

Risk factors for being a Long drainer were analyzed and are summarized in Table 3. For relative risk evaluation, we considered cutoff values of 28 kg/m^2^ for body mass index and 750 g for specimen weight.

**Table 3:** Risk Factors for Long drainers (6 days or more with drains)

				Logistic regression		
		Short drainers	Long drainers	Relative risk	(95% CI)	*P value*
BMI, kg/m^2^	< 28	190 (73.1%)	70 (26.9%)			
Total number (%)	≥ 28	72 (47.7%)	79 (52.3%)	2.98	(1.96–4.54)	0.001
Arterial hypertension	No	242 (65.2%)	129 (34.8%)			
Total number (%)	Yes	25 (53.2%)	22(46.8%)	1.65	(0.90–3.04)	0.197
Diabetes mellitus	No	259 (64.8%)	141(35.3%)			
Total number (%)	Yes	8(44.4%)	10 (55.6%)	2.30	(0.89–5.95)	0.151
Previous bariatric surgery	No	249 (66.2%)	127 (33.8%)			
Total number (%)	Yes	18 (42.9%)	24 (57.1%)	2.61	(1.37–4.99)	0.005
Previous abdominal surgery	No	91 (67.4%)	44 (32.6%)			
Total number (%)	Yes	176 (62.2%)	107 (37.8%)	1.26	(0.82–1.94)	0.855
Specimen weight, g	< 750 g	66 (83.5%)	66 (83.5%)			
Total number (%)	≥ 750 g	186 (59.4%)	127 (40.6%)	3.48	(1.84–6.55)	0.000

Short drainers (less than 6 days with drains; *n* = 267); Long drainers (6 days or more with drains; *n* = 151)

BMI, body mass index

For the variables, body mass index and specimen weight cutoffs of 28 kg/m^2^ and 750 g were considered, respectively

Previous bariatric surgery, body mass index ≥ 28 kg/m^2^, and specimen weight ≥ 750 g were revealed to be the variables with a higher increase in the risk for being a Long drainer. Long drainers were 2.6 times more likely to be patients with previous bariatric surgery. Long drainers were 3.0 times more likely to have a body mass index ≥ 28 kg/m^2^ than a lower body mass. Specimen weight was the highest risk factor for being a Long drainer, as the latter was 3.5 times more likely to occur with specimen weight ≥ 750 g.

**Fig. 1:**
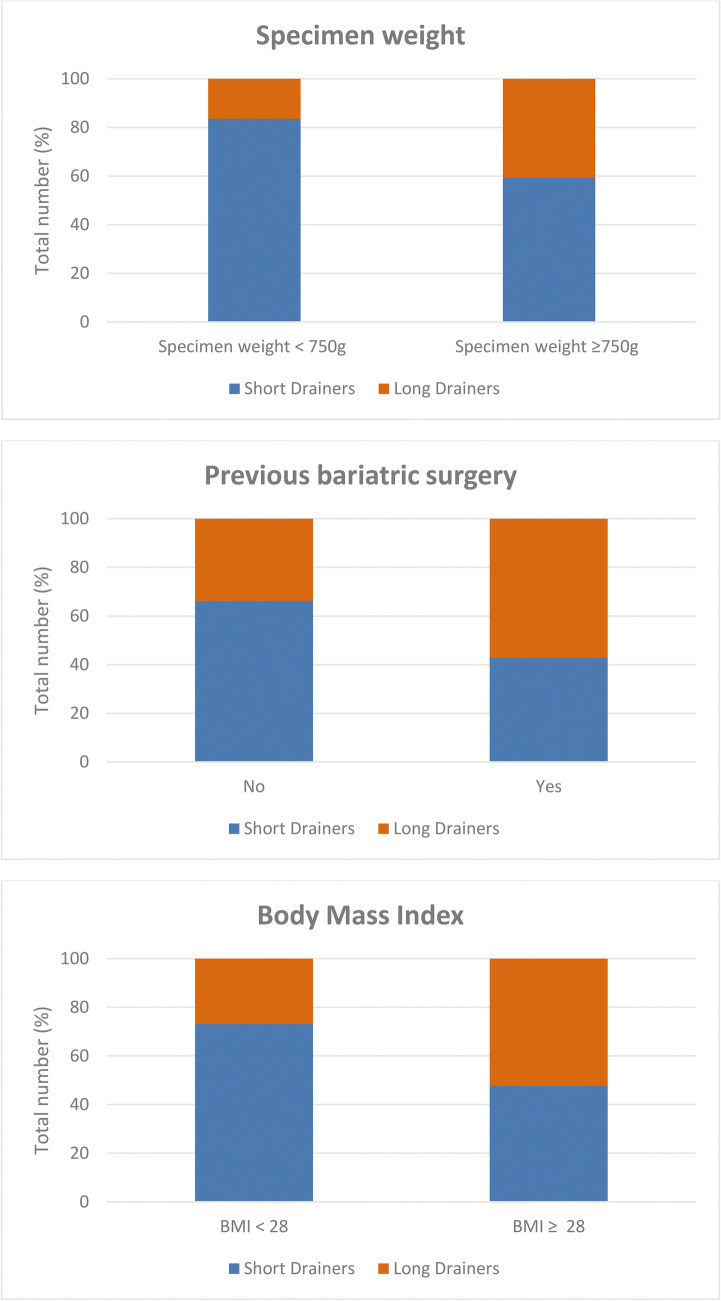
Distribution of the specimen weight, previous bariatric surgery, and body mass index variables between Short (< 6 days; *n* = 267) and Long drainers (≥ 6 days; *n* = 151)

The principal component analysis concluded that the variables mean specimen weight ≥ 750 g, body mass index ≥ 28 kg/m^2^, and previous bariatric surgery were responsible for 75% of Long drainers obtained in this study. Figure 1 shows the distribution of Long and Short drainers between these variables. Considering patients with a specimen weight ≥ 750 g, 40.6% were Long Drainers. A very different reality was observed for patients with specimen weights lower than 750 g, as Long drainers were present in 16.5%. Patients previously submitted to bariatric surgery were Long drainers in 57.1 %, while patients without bariatric procedures were Long drainers in 33.8 %. 52.3% of the patients with a body mass index ≥ 28 kg/m^2^ were Long drainers compared to 26.9% in the group with a lower body mass index.

## Discussion

This retrospective observational study provides evidence that some patients’ characteristics and risk factors enhance the probability of being a Long drainer, with five clinically important and statistically significant results:

- Specimen weight and body mass index are higher in Long drainers, and previous bariatric surgery is more prevalent;
- Specimen weight ≥ 750 g increases the risk by 3.5 times;
- Body mass index ≥ 28 kg/m^2^ increases the risk by 3.0 times;
- Previous bariatric surgery increases the risk by 2.6 times;
- Specimen weight ≥ 750 g, body mass index ≥ 28 kg/m^2^, and previous bariatric surgery are responsible for 75% of Long drainers.

No significant differences were found in age, sex, arterial hypertension, *diabetes mellitus*, and previous abdominal surgeries, pointed out as risk factors for abdominoplasty complications in previous studies [15, 16].

Two recently introduced innovations in abdominoplasty, Scarpa fascia preservation, and progressive tension “quilting” sutures are effective for drain use reduction or elimination [10, 11, 17–27]. Another possible surgical strategy to achieve this goal is related to the dissection technique [28–30]. Nevertheless, drains are probably the most frequently used method for decreasing complications after a full abdominoplasty [6, 8]. Drains are usually removed based on volumetric criteria, and a drain output in each drain less than or equal to 30ml collected over 24 h seems to be the more consensual [5, 8]. Applying these criteria, it is possible to observe patients with drain usage higher than six days, for whom we proposed the denomination of Long drainers. Previously, we reported a 33% incidence of Long drainers in a non-bariatric population and 52% in a bariatric population, both submitted to classical abdominoplasty [10, 11, 31]. The present study is in accordance with these previous findings, as 36% of the patients in the current study were Long drainers. Long periods with drains are undesirable and may result in slower recovery and patient discomfort.

Previous studies suggested a strong correlation between the amount of tissue removed and postoperative overall complications in abdominoplasty [32, 33]. Shermak [33] concluded that the most important risk factor for seroma is the weight of skin excised during surgery. In the current study, the mean specimen weight was the most determinant factor for being a Long drainer. Long drainers had an average weight of the tissue removed of 1568 g, 40% higher than those found in Short drainers.

The influence of patient body mass index in abdominoplasty complication rates is well known [1, 9, 15, 32, 34, 35]. Vastine [34] and Neaman [32] reported a significantly increased complication rate of 80% and 76.9% in overweight patients, respectively, with patient obesity adversely affecting the surgical results. Kim [9] verified that patients with a body mass index greater or equal to 25.0 kg/m^2^ were more likely to develop seromas and have long periods with drains than patients with average weight (< 25 kg/m^2^). In the present study, body mass index was the second risk factor with more influence on days with drains, and patients with body mass index ≥ 28 kg/m^2^ had 3.0 times more risk of being a Long drainer.

Nowadays, an ever-increasing number of post-bariatric patients are being admitted for abdominoplasty. This set of patients reveals a higher complication rate after surgery, with more prolonged use of drains [5, 11, 31, 32]. As we have already pointed out, obesity is a well-recognized risk factor in body contour surgery, particularly abdominoplasty. This risk persists even after massive weight reduction [36]. Several factors have been suggested to explain this, such as tissue [37–39] and metabolism changes [40]. Also, prior higher body mass index and weight loss with more significant deformities require more extensive operations, longer incisions, and area of removed tissue [41]. Two previous investigations have shown a very high incidence of Long drainers in bariatric patients submitted to classical abdominoplasty, specifically 52% and 56% [11, 31]. In concordance with published data, the prevalence of previous bariatric surgery in our patients was significantly higher in Long drainers than in Short drainers (15.9% vs 6.7%). It was the third risk factor that had the most impact on Long drainers’ clinical profile.

Comorbidities, like *diabetes mellitus* and hypertension, are frequently related to increased complications when performing abdominoplasty [15, 32]. *Diabetes mellitus* influence in plastic surgery procedures was found to have more impact on body contour procedures, specifically abdominoplasty [42]. Although we verified an increased contribution for Long drainers of *diabetes mellitus* and hypertension, no statistical differences were found between Short and Long drainers. It is essential to highlight that our department policy considers strict control of these chronic diseases mandatory for safely performing abdominoplasty.

Although a history of multiple abdominal procedures has been suggested as an eventual risk factor [15], our results revealed that it presented the lowest influence in the Long drainer profile.

No statistical differences concerning age and sex were found in this study, in contrast to others that showed complication rates significantly higher for patients aged 60 years or older [15, 32] and for male sex [1, 15].

The present study has important strengths that must be pointed out: It includes a population of more than four hundred patients from a single center, analyzing patients’ characteristics and risk factors that demonstrated to impact volume and days with drains when performing an abdominoplasty. Nevertheless, it has some limitations that have to be mentioned. Its retrospective character arises as the main limitation. Furthermore, the five surgical teams involved can represent an element of bias. Some limitations could be overcome in a prospective study involving only one surgeon. It is also important to highlight that most patients involved in the current study were females; thus, the findings apply to women. Further studies are needed for male patients. The current study was done with patients submitted to a classical abdominoplasty technique, so they do not apply to more recent surgical strategies such as quilting sutures or Scarpa sparing abdominoplasty.

Nevertheless, the current study identified risk factors for being a Long drainer. A careful assessment of these variables can help the surgeon screen Long drainers among the proposed abdominoplasty candidates and counsel them on the attendant risks of surgery. When these variables are present, the surgeon should consider using efficient surgical strategies for shortening drain volume and time to drain removal. Scarpa fascia preservation is one of these strategies developed over the years [43], and it is one of the distinctive components of Saldanha’s well-known lipoabdominoplasty technique [44–48]. Studies have shown that preserving Scarpa fascia during an abdominoplasty improves recovery by reducing several factors, namely total and daily drain output, time to drain removal, and seroma [11, 21, 49]. Long drainers were reduced from 33 to 1% using abdominoplasty with Scarpa fascia preservation [2, 10, 21]. These advantages have also been confirmed in the bariatric population and have had a significant impact. The high-risk bariatric patients had similar postoperative recovery and outcomes to non-bariatric patients, presenting a substantial reduction in Long drainers [11, 31].

Shortening time with drains should be a concern and a goal for any surgeon performing an abdominoplasty. Patients will always benefit from that strategy. The results of our study should be considered by any surgeon aiming at drainless abdominoplasty for adequate selection of a reasonable candidate. The Long drainer high-risk clinical profile identified in the current study may hinder that goal, i.e., drainless abdominoplasty.

Concerning the external validity and applicability of our findings, we would like to clarify that the policy in our department is to propose a full abdominoplasty for patients presenting with abdominal deformities with excess skin and adiposity along with muscle laxity (Bozola and Psillakis types III and IV [50] and Matarasso Types III and IV [51]). Patients with a BMI higher than 30 are not considered candidates for a full abdominoplasty except if they had been previously submitted to bariatric surgery. The clinical series analyzed in this research excluded *fleur-de-lis* abdominoplasty, lower body lift, mini-abdominoplasty, and lipoabdominoplasty. The cutoffs for surgical specimen weight and body mass index were chosen according to average values in our current practice. Another aspect to clarify is that the policy in our department is to discharge abdominoplasty patients after drain removal. Inpatient abdominoplasty was favored based on closer monitoring of pain, hydration, daily drain output, and any complication related to the drainage vacuum system. It is also easier to adjust and ensure compliance with medication and early ambulation. Feedback from our patients indicated that this strategy improves their comfort because they are discharged without drains. Nevertheless, our findings may apply to other approaches, such as abdominoplasty performed as an outpatient procedure.

## Conclusion

This is the first study analyzing the clinical profile of patients who had to use suction drains for long periods after a classical full abdominoplasty. Some risk factors for being a Long drainer have been identified: body mass index ≥ 28 kg/m^2^, previous bariatric surgery, and specimen weight ≥ 750 g. These three factors were responsible for 75% of Long drainers in the current study. Their presence may justify the use of surgical strategies for Long drainer prevention, such as progressive tension sutures or Scarpa sparing abdominoplasty.
